# In Vitro Establishment and Maintenance of Culture Lines with Differentiated Somatic Embryogenesis Capacity in Olive (*Olea europaea* L.)

**DOI:** 10.3390/plants14182881

**Published:** 2025-09-16

**Authors:** Rita Pires, Hélia Cardoso, Lénia Rodrigues, Augusto Peixe

**Affiliations:** 1 MED (Mediterranean Institute for Agriculture, Environment and Development) & CHANGE—Global Change and Sustainability Institute, IIFA (Institute for Research and Advanced Training), University of Évora, Mitra Campus, Ap. 94, 7002-554 Évora, Portugal; liar@uevora.pt; 2 MED (Mediterranean Institute for Agriculture, Environment and Development) & CHANGE—Global Change and Sustainability Institute, Department of Biology, School of Science and Technology, University of Évora, Mitra Campus, Ap. 94, 7002-554 Évora, Portugal; hcardoso@uevora.pt; 3 MED (Mediterranean Institute for Agriculture, Environment and Development) & CHANGE—Global Change and Sustainability Institute, Department of Plant Science, School of Science and Technology, University of Évora, Mitra Campus, Ap. 94, 7002-554 Évora, Portugal

**Keywords:** somatic embryogenesis, *Olea europaea*, cyclic embryogenesis, embryogenic lines, conversion

## Abstract

Somatic embryogenesis (SE) is a powerful biotechnological tool for large-scale clonal plant propagation. However, most woody species exhibit a recalcitrant response. *Olea europaea* L., a highly valuable tree crop, is among these recalcitrant species. Unravelling the molecular mechanisms underlying SE efficiency in *O. europaea* is, therefore, essential. Access to embryogenic lines with contrasting capacities for somatic embryo differentiation is a key requirement for such studies. Mature fruits of olive cultivars ‘Galega Vulgar’ and ‘Arbequina’ were collected from open-pollinated orchard-grown plants, and various explants taken from zygotic embryos were used to establish SE. A differentiated response was observed both within each cultivar and between cultivars, with cv. ‘Arbequina’ showing the highest embryogenic induction, particularly when radicles were used. Secondary SE was successfully established for both high- and low-efficiency lines, providing valuable material for future molecular studies. Somatic embryo conversion into plants, a key indicator of embryo quality, was successfully achieved in both cultivars. Flow cytometry analysis revealed a high degree of chromosomal stability. This study presents a reliable procedure to obtain and maintain distinct embryogenic responses in *O. europaea*, identifying lines with both high and low embryogenic efficiency that can serve as model systems for future molecular investigations.

## 1. Introduction

Somatic embryogenesis (SE) is an in vitro technique in which asexual embryos arise from cells of a tissue or an organ, enabling the regeneration of a complete plant [1]. SE presents several applications, including the large-scale propagation of elite genotypes, the production of synthetic seeds for germplasm preservation, and in vitro plant regeneration to support breeding programs [2]. The capacity to switch the developmental pathway of cells from vegetative to embryogenic, thereby generating somatic embryos, is restricted to a limited subset of cells and is affected by several endogenous and exogenous factors [3,4]. Plant genotype is considered one of the most important factors [5]. Even within the same species, cultivars and even individual plants within a cultivar can show considerable variation in their ability to initiate and develop somatic embryos. *Vitis vinifera* [6], *Cocos nucifera* [7], *Theobroma cacao* [8], and *Physocarpus opulifolius* [9] are examples where a strong effect of genotype has been reported, highlighting the need to establish a protocol for each new cultivar within a species.

Understanding the sequential stages of SE is essential, as the success of an SE protocol relies on the ability of the initial explants to progress through the phases of induction, expression, maturation, and germination [10]. The induction phase, commonly characterised as a stress-related response [11], involves the perception and activation of a complex signalling network, which, in turn, modulates the expression of target genes [12,13]. Besides genotype dependency, factors such as explant type, hormone balance, and culture conditions strongly influence the efficiency of this phase [14]. In olive (*Olea europaea* L.), SE is neither routinely nor widely applied, largely because of its strong genotype dependence [2], and its frequent recalcitrant behaviour in response to in vitro protocols [15]. This limitation is particularly evident when attempting to induce SE using adult tissues as the initial explant, although successful cases have been reported in a few cultivars such as ‘Canino’ and ‘Moraiolo’ [15], ‘Chetoui’ [16], ‘Dahbia’ [17], ‘Picual’ [18], and ‘Moroccan Picholine’ [19].

The expression and maturation phases are characterised by embryo differentiation and key morphological changes [20,21,22], including cell elongation, dehydration, and the accumulation of storage compounds such as carbohydrates, lipids, and proteins [23,24]. Therefore, physiological maturation is essential for transition to germination, the final phase in which somatic embryos develop into complete plantlets [23,25]. Low germination rates remain a major constraint for large-scale SE propagation in olive. Key factors affecting embryo germination include the composition of the culture medium [26,27], the use of osmotic agents [28], environmental growth conditions [29,30,31,32,33], and the presence of abnormal embryos [34]. The differentiation of abnormal embryos and the appearance of somaclonal variations have been attributed to multiple factors, including genetic stability and the occurrence of epigenetic events [35,36,37,38], with a strong dependence on the genotype and the duration of culture maintenance [30]. Studies conducted on the olive cv. ‘Picual’ were verified using flow cytometry and molecular markers to establish the link between somaclonal variations and long-term cultures [30,39].

In secondary SE, the initiation of new embryos from primary somatic embryos [40] allows for the long-term maintenance and proliferation of embryogenic cultures [2,40]. Although morphogenic capacity may decline as a result of somaclonal variation, culture ageing, or repeated subculturing [35,36,37], secondary SE effectively supports high-frequency plant regeneration in species such as *Vitis* spp. [41], *Rosa hybrida* [42], *Quercus* spp. [43,44], and *Centaurium erythraea* [40]. By ensuring consistent proliferation, uniform cultures, and sustained embryogenic competence, secondary SE represents a reliable strategy for the long-term maintenance of productive clonal lines [21,40]. Although a gelled medium is used in the majority of studies on secondary SE in olive [28,29,31,45], a liquid culture may provide a more favourable environment to regulate cluster interaction, nutrient uptake, and cell proliferation [46]. Moreover, this system enables the identification and establishment of embryogenic lines with distinct capacities by differentiating morphogenic clusters in suspension cultures based on their morphology and gene expression patterns [47,48].

In the study presented here, an efficient method is described to establish *O. europaea* embryogenic lines with different embryogenic capacities through secondary SE in a liquid culture medium using the Portuguese olive cv. ‘Galega Vulgar’ and the Spanish cv. ‘Arbequina’. The germination ability of these long-term cultures, as well as the genetic stability of embryos and plantlets, was also investigated. This approach enables the selection of both high- and low-efficiency embryogenic lines, providing a platform for future research into the molecular mechanisms regulating SE efficiency in olive.

## 2. Results

### 2.1. Establishment of Somatic Embryogenesis

The rate of callogenesis and rhizogenesis, as assessed 21 days after explant establishment in the induction medium, indicates *calli* development in all types of explants (radicles, proximal, and distal cotyledons) in both cultivars. Significant differences were observed in explants from cv. ‘Galega Vulgar’, with radicles exhibiting the highest callogenesis rate (Table 1). In contrast, cv. ‘Arbequina’ showed the highest average callogenesis rate (approximately 70%) and significantly higher rates than cv. ‘Galega Vulgar’. No significant differences were observed among the different explant types. The development of adventitious roots (rizhogenesis) was observed in all explants in both cultivars, with cv. ‘Arbequina’ exhibiting significantly higher rates compared to cv. ‘Galega Vulgar’. Despite the overall higher rhizogenesis in cv. ‘Arbequina’, no significant differences were found among the three explant types within this cultivar. In contrast, in cv. ‘Galega Vulgar’, the radicle exhibited a significantly higher rhizogenesis rate compared to the proximal and distal regions of cotyledons (see Table 1).

**Figure 1:**
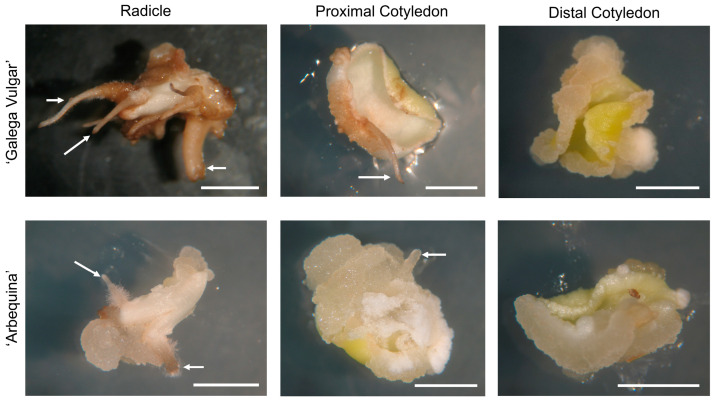
Aspect of the *calli* achieved in all types of initial explants after 21 days in induction OMc medium. The presence of adventitious roots is highlighted with white arrows. Bars: 0.5 cm.

The *calli* morphology formed at the end of the induction phase showed morphological uniformity across all explants. The obtained *calli* were predominantly formed on the periphery of the explants at the wound regions, exhibiting a colour ranging from pale yellow to white and friable consistency (Figure 1).

All the explants that responded to embryogenic induction were transferred into an OMc gelled expression medium to promote embryo differentiation. After 30 days under expression conditions, the embryogenic structures were observed on the surface of the *calli* and often underneath them, staying in direct contact with the culture medium.

At the expression phase, the percentage of explant necrosis and the percentage of explants that responded positively to SE are presented in Table 1. In both the proximal and distal cotyledons, the overall rate of necrosis during the expression phase was substantially higher in cv. ‘Arbequina’ than in cv. ‘Galega Vulgar’ (Table 1). In cv. ‘Galega Vulgar’, significant differences were observed among explants, with radicles showing the highest necrosis rate.

The capacity of the explants to respond to the stimulus of SE can also be observed in Table 1. The radicle exhibited the highest response rate to the SE stimulus when compared to the proximal and distal cotyledons; however, significant differences among explants were only detected in cv. ‘Arbequina’. Proximal cotyledons exhibited a significantly lower number of explants developing somatic embryos, although no significant differences were observed between distal cotyledons and radicles.

To promote the proliferation of embryogenic *calli*, all embryogenic plant material (embryogenic *calli* and somatic embryos at early developmental phases) was transferred to the ECO gelled medium for two subcultures. At the end of the second subculture, explants were transferred to the ECO liquid culture medium for three additional subcultures.

### 2.2. Characterisation of the Embryogenic Lines Established Under Liquid Medium

**Figure 2:**
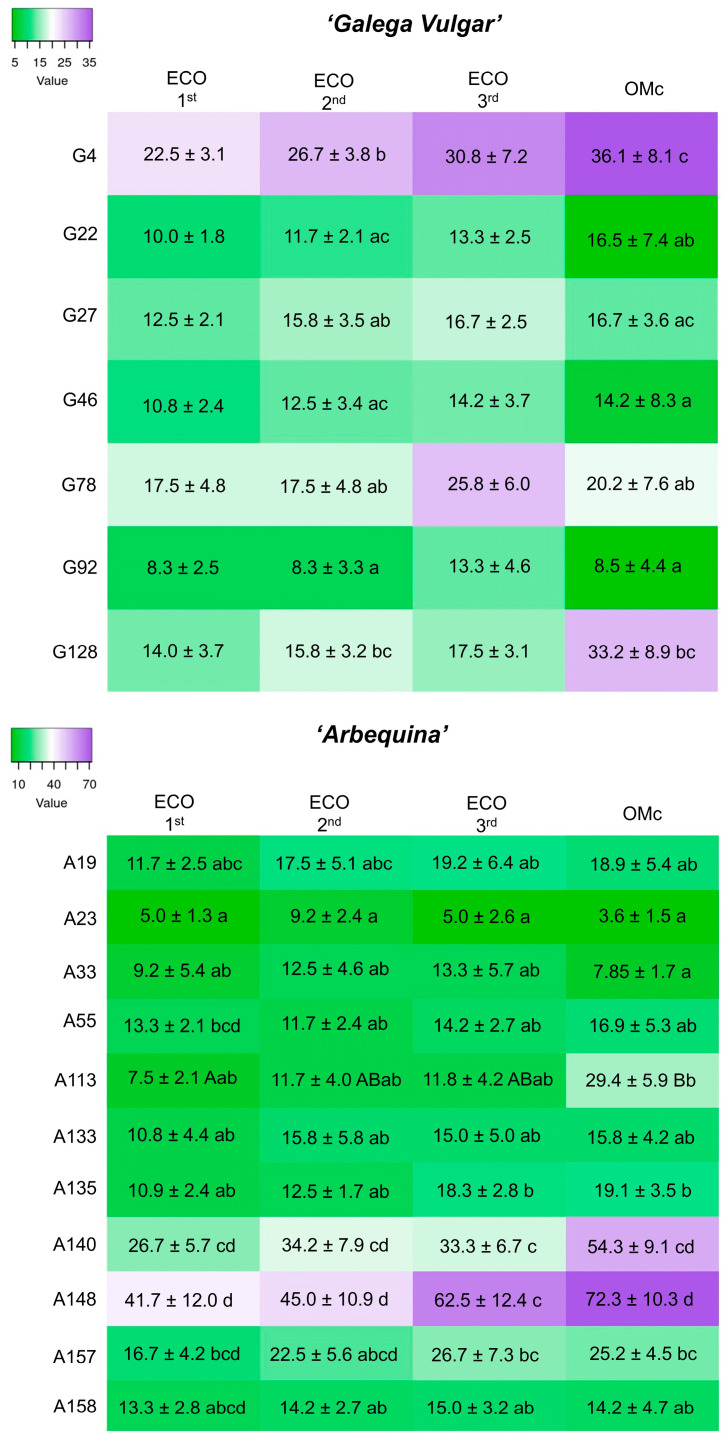
Number of somatic embryos per gram of *calli* of all embryogenic lines in the two cultivars under evaluation (cv. ‘Galega Vulgar’ and cv. ‘Arbequina’) during three subcultures in ECO liquid medium and one subculture in OMc expression medium. Different lowercase letters indicate statistically significant differences (*p* ≤ 0.05) between lines, while capital letters represent differences between subcultures. Data are presented as the mean ± standard error.

To evaluate the embryogenic potential of the different embryogenic lines established in liquid medium, 200 mg of embryogenic tissue from each line was inoculated in ECO liquid medium and maintained through three consecutive subcultures, followed by a final transfer to OMc expression medium to further evaluate the embryo conversion rate. Each subculture had a duration of 4 weeks, after which the number of somatic embryos formed per gram of *calli* was recorded (Figure 2). Initially, eleven embryogenic lines of the cv. ‘Galega Vulgar’ and twenty lines of cv. ‘Arbequina’ were established. However, not all lines exhibited sustained viability under liquid culture conditions. Several showed progressive oxidation and loss of morphogenic potential, leading to their exclusion from the experiment. Ultimately, seven embryogenic lines of cv. ‘Galega Vulgar’ and eleven of cv. ‘Arbequina’ were processed for subsequent stages of the assay.

Within each embryogenic line, the first subculture in ECO liquid medium produced fewer somatic embryos, with numbers progressively increasing in subsequent subcultures and, in most cases, reaching the highest rates in the third subculture (exceptions were observed in lines A23, A133, and A140). Regarding the transfer to OMc expression medium, not all *calli* responded with an increase in SE efficiency. While some lines showed an increase in the number of differentiated embryos, others remained stable, and some exhibited a slight decrease (e.g., G78 and G92; A19, A23, A33, A157, and A158). Nevertheless, these changes were only statistically significant in line A113 (*p* = 0.042).

A comparison of somatic embryo production across the embryogenic lines of each cultivar was also conducted within each subculture. During the first subculture in ECO liquid medium, no significant differences between the embryogenic lines in the cv. ‘Galega Vulgar’ were found (*p* = 0.073); however, line G4 displayed a greater number of somatic embryos. In fact, across the different subcultures, G4 consistently showed the greatest capacity for somatic embryo formation, with significant differences emerging from the first subculture onwards, except in comparison with line G128. Lines G22 and G92 consistently produced the lowest number of somatic embryos (Figure 2). In cv. ‘Arbequina’, lines A140 and A148 produced the highest number of somatic embryos across all subcultures. In contrast, lines A10, A23, A33, and A158 showed the lowest embryo production, with line A23 consistently exhibiting the lowest values across all subcultures. Similar to what was observed in cv. ‘Galega Vulgar’, some lines (A19, A23, A33, A157, and A158) produced a higher number of somatic embryos during the third subculture in ECO liquid medium, although these differences were not statistically significant.

**Figure 3:**
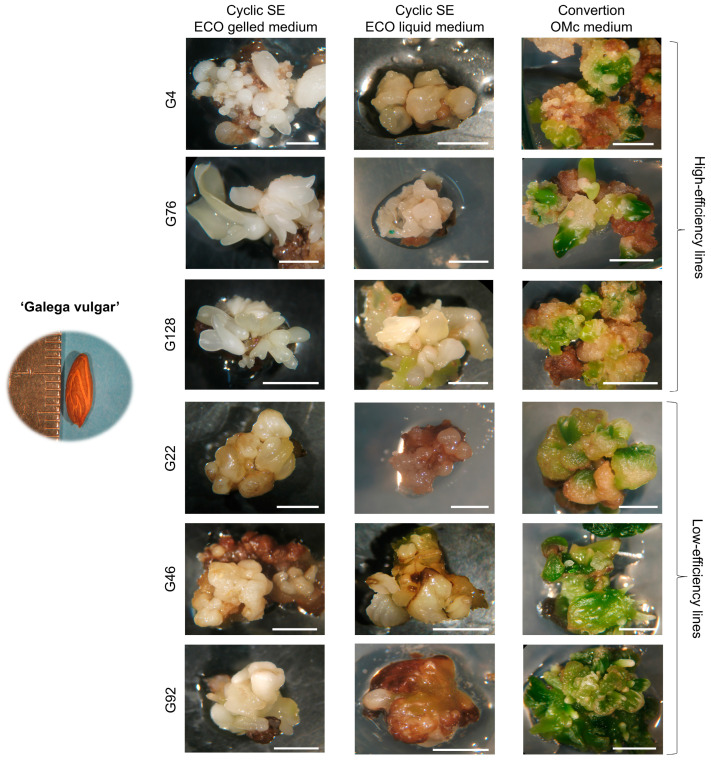
Embryogenic *calli* from explants derived from the cv. ‘Galega Vulgar’ in cyclic SE ECO (gelled and liquid medium) and in the OMc expression medium. Bars: 0.5 cm.

**Figure 4:**
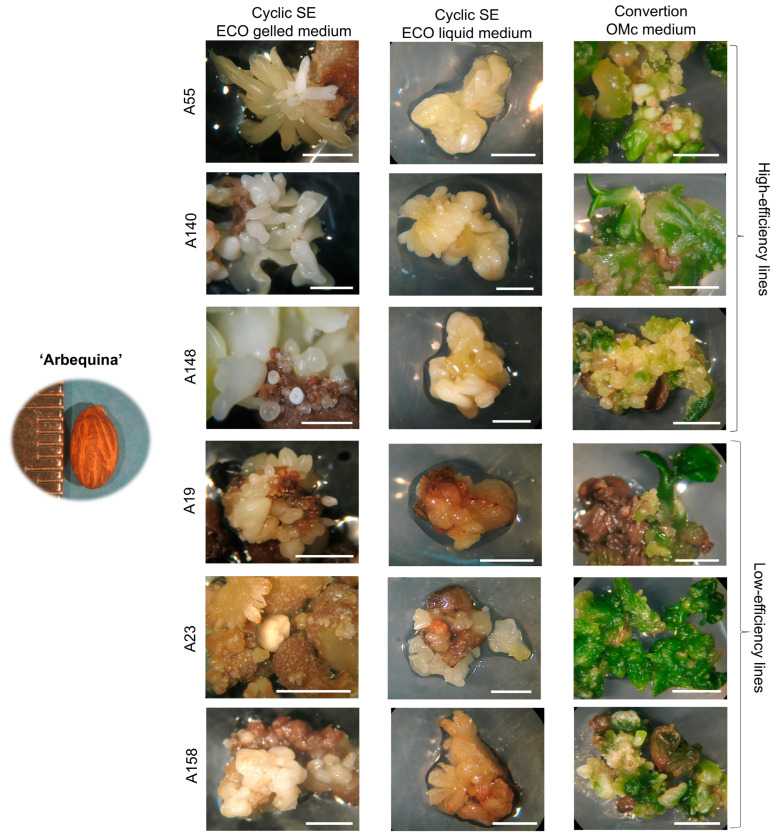
Embryogenic *calli* from explants derived from the cv. ‘Arbequina’ in cyclic SE ECO (gelled and liquid medium) and in the OMc expression medium of somatic lines. Bars: 0.5 cm.

Morphological differences in *calli* structure were consistently observed between high- and low-efficiency embryogenic lines in both cultivars. Differences between embryogenic lines are illustrated in Figure 3 and Figure 4, which show representative images at the end of the second subculture in ECO gelled medium, after the third subculture in ECO liquid medium, and following transfer to OMc expression gelled medium in both cultivars. In ‘Galega Vulgar’ (Figure 3), high-efficiency lines (G4, G76, G128) developed fragmented *calli* with abundant globular-stage somatic embryos. In contrast, low-efficiency lines (G22, G46, G92) produced large compact *calli* with markedly fewer embryos.

Similar results were observed in explants derived from cv. ‘Arbequina’ (Figure 4). High-efficiency lines produced friable and well-organised *calli*, frequently associated with abundant somatic embryos, particularly at the globular stage. In contrast, low-efficiency lines (A19, A23, A158) developed dense compact *calli* with reduced embryogenic capacity and a higher incidence of necrosis throughout successive subcultures. After the embryogenic *calli* were transferred to the OMc expression medium under light, this reduction became more pronounced. While increased somatic embryo production was observed in some lines of cv. ‘Arbequina’ (A113, A133, A135, A140, A148) and cv. ‘Galega Vulgar’ (G4, G22, G128), other lines showed the opposite trend. After being transferred to expression medium, lines A19, A23, A33, A157, and A158 from cv. ‘Arbequina’ and G46, G78, and G92 from cv. ‘Galega Vulgar’ showed a decrease in somatic embryo production.

The high- and low-efficiency embryogenic lines presented in Figure 3 and Figure 4 were selected for plant conversion assessment after subculture in OMc expression medium. Classification was based on consistent differences observed throughout the subculture period, including the average number of somatic embryos formed per mg of *calli*, regenerative capacity of the *calli*, and incidence of tissue necrosis. High-efficiency lines consistently exhibited greater embryo production, enhanced regeneration, and reduced necrosis.

### 2.3. Conversion of Somatic Embryos

High- and low-efficiency embryogenic lines were analysed to determine which were the most suitable for embryo conversion into plants. Following the procedure outlined by Pires et al. [31], embryos at the globular and torpedo stages were individually collected and inoculated onto OMc expression medium for 30 days. At the end of this period, the number of converted somatic embryos was recorded. The cotyledonary stage was not considered, as most of the somatic embryos observed at this stage exhibited morphological anomalies.

**Figure 5:**
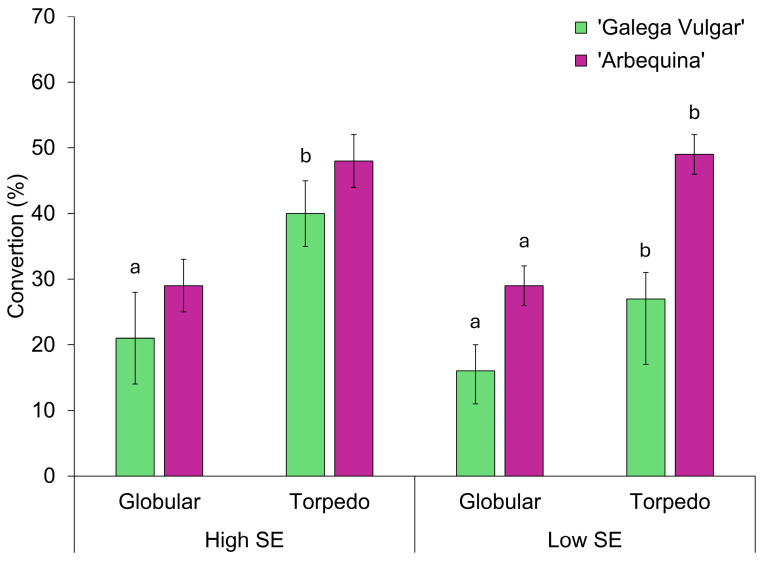
Conversion rates of somatic embryos collected at different developmental stages from high- and low-efficiency embryogenic lines (mean ± standard error) of both cultivars. Statistically significant differences between stages are denoted by distinct lowercase letters. Statistical significance was set at *p* ≤ 0.05.

Regarding embryo conversion, somatic embryos from high-efficiency lines yielded results comparable to those from low-efficiency lines (Figure 5). In cv. ‘Galega Vulgar’, high-efficiency lines converted more somatic embryos into plants; however, no significant differences were observed compared to low-efficiency lines. Although the torpedo stage resulted in a significantly higher conversion rate of somatic embryos into plants in both cultivars, no significant differences were observed among developmental stages in high-efficiency lines of cv. ‘Arbequina’.

**Figure 6:**
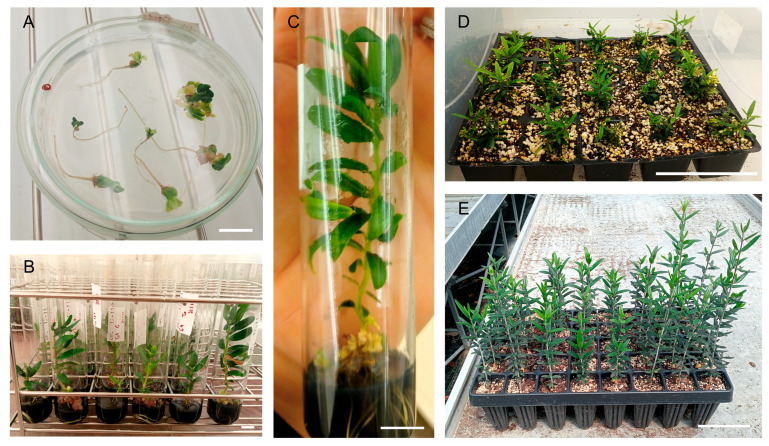
Converted somatic embryos of cv. ‘Galega Vulgar’ and cv. ‘Arbequina’, and plants at various developmental stages, including acclimatised plants. (**A**) Somatic embryos at the end of the 30-day conversion period. (**B**,**C**) Converted somatic embryos of cv. ‘Galega Vulgar’. (**D**) Acclimatised plants derived from somatic embryos of cv. ‘Arbequina’. (**E**) Acclimatised plants derived from somatic embryos of cv. ‘Galega Vulgar’. Bars for (**A**–**C**) correspond to 1 cm, and, for (**D**,**E**), 10 cm.

All somatic embryos, including those exhibiting morphological abnormalities (Figure 6A), were transferred to test tubes containing OMc expression medium supplemented with activated charcoal (Figure 6B,C). After 4 weeks, leaf samples were collected from plants regenerated from both normal and abnormal somatic embryos to enable flow cytometric analysis for the detection of ploidy level variations.

The plants that developed from normal embryos of both cultivars were successfully acclimated under the conditions employed, regardless of the fact that the study did not take the embryos’ acclimatisation process into account (Figure 6D,E).

### 2.4. Histological Observations

**Figure 7:**
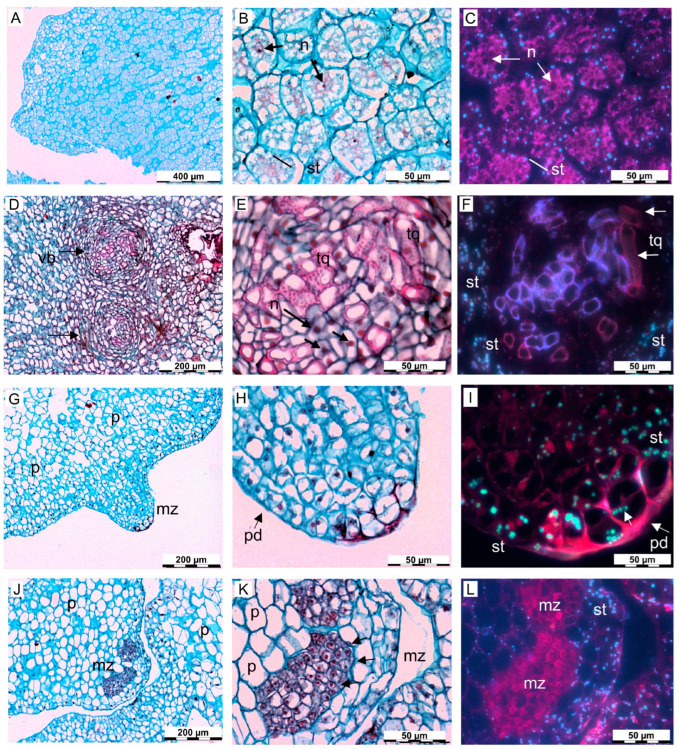
Histological observations of high- (**A**–**F**) and low-efficiency (**G**–**L**) embryogenic *calli.* (**A**) Overview of a high-efficiency *calli* showing compact and well-organised cellular structure. (**B**,**C**) High-efficiency *calli* with prominent nuclei (n) and abundant starch deposits (st) under light and fluorescence microscopy, respectively. (**D**–**F**) Somatic embryo primordia in high-efficiency *calli* with clear vascular bundle formation (vb), including concentric xylem and phloem arrangement, and differentiated tracheids (tq) with lignified walls, highlighted by fluorescence (**F**). (**G**–**I**) Low-efficiency *calli* displaying loosely arranged parenchyma cells (p) and meristematic zones (mz) located peripherally. Cells exhibit reduced cytoplasmic content and lower starch accumulation, with protodermal organisation (pd). (**J**–**L**) Low-efficiency *calli* displaying meristematic zones (mz) with small actively dividing cells. Abbreviations: mz, meristematic zone; n, nucleus; p, parenchyma; pd, protodermis; st, starch; tq, tracheid; vb, vascular bundle.

Histological observations were conducted on high- and low-efficiency lines collected at the end of the third subculture in ECO liquid medium (Figure 7). Clear cytological differences were consistently observed between the two types. In high-efficiency lines, embryogenic tissues were mainly composed of isodiametric parenchyma cells, with isolated proembryonal masses originating from the epidermal layer (Figure 7A,C). These cells exhibited dense cytoplasm, a high nucleus-to-cytoplasm ratio, and well-defined cell walls. Frequent periclinal and anticlinal divisions of epidermal cells were also noted (Figure 7C). Starch granules were detected in both line types, particularly in epidermal and sub-epidermal layers, but were absent in zones of active cell division (Figure 7C,F,I,L). Embryogenic cells in high-efficiency lines formed structured clusters or meristematic zones, rarely observed in low-efficiency *calli*. Additionally, the vascular bundles were seen to be arranged in a concentric arrangement, with phloem situated peripherally and xylem in the inner regions (Figure 7D–F). Tracheids within these bundles showed elongated forms with thick lignified cell walls, dark-pink staining, and lighter lumens (Figure 7E); these structures were more evident under fluorescence microscopy (Figure 7F).

In contrast, low-efficiency lines (Figure 7G–L) exhibited less organised cellular architecture and tissue structure compared to high-efficiency lines, characterised by larger vacuolated cells, reduced cellular density, and meristematic zones confined to specific peripheral regions of the explants, indicating a loss of embryogenic competence (Figure 7G,J). These regions, characterised by higher cell density, contained clusters of embryonic cells arranged in layers or small groups. Cells in low-efficiency lines were generally larger, with less abundant cytoplasmic content. Although starch granules were visible in epidermis-like parenchyma cells, they were less abundant than in high-efficiency tissues.

In summary, histological analysis revealed clear structural differences between high- and low-efficiency embryogenic *calli*. High-efficiency lines displayed organised cell layers, high cytoplasmic content, abundant starch, and active somatic embryo primordia often associated with vascular bundle formation. Low-efficiency lines, on the other hand, displayed less distinct embryogenic zones, loosely arranged parenchyma cells, and fewer metabolic indicators. These results support the relevance of cellular architecture as a reliable marker of embryogenic potential and line efficiency.

### 2.5. Flow Cytometry

**Figure 8:**
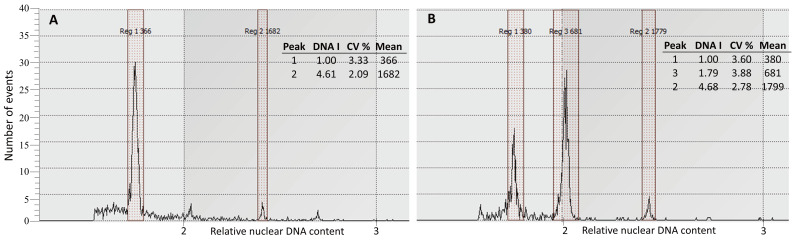
Estimation of absolute nuclear DNA content in somatic embryogenic *calli*. The G1 peak of *Olea europaea* is indicated as Reg 1, while the G1 peak of the standard sample (*Allium schoenoprasum*) is indicated as Reg 2. The DNA histograms show both G1 and G2/M peaks. Panel (**A**) represents samples with stable diploid profiles (2×), while Panel (**B**) illustrates a sample with altered DNA content, suggesting chromosomal instability, as evidenced by the presence of additional or shifted peaks.

Flow cytometry analysis of 45 samples across embryogenic lines revealed consistent ploidy levels among the different tissue types, including high- and low-efficiency embryogenic *calli*, abnormal somatic embryos, and leaf samples taken from normal and abnormal converted embryos. All samples displayed DNA content peaks corresponding to the diploid reference species *Allium schoenoprasum*, indicating the maintenance of diploidy throughout SE and plant conversion. The results showed that 93.4% of the analysed samples exhibited the normal diploid level for olive (2×), as exemplified in Figure 8A. Nevertheless, in 6.6% of the samples, variations in the relative DNA ratio were identified, as represented in Figure 8B, where high-efficiency embryogenic calli presented 75% of the cells as diploid, and 25% exhibited aneuploidy, indicating chromosomal instability. The presence of a more pronounced intermediate peak suggests a higher proportion of cells in the S phase, indicating a higher rate of cell proliferation in this line. Furthermore, the CV% values were slightly higher, particularly in the intermediate peak (2.78%), reflecting greater heterogeneity in DNA replication.

Flow cytometry analysis of abnormal somatic embryos identified in cv. ‘Galega Vulgar’ and cv. ‘Arbequina’ revealed that all samples were diploid (2×), indicating that the observed morphological abnormalities were not associated with changes in ploidy. This suggests that other factors, possibly related to environmental stress or genetic factors, may be contributing to the observed somatic embryo abnormalities. Leaves from regenerated plants exhibited stable diploid profiles, confirming that the SE process and plant regeneration maintained chromosomal integrity.

## 3. Discussion

Mature zygotic embryos from two olive cultivars (cv. ‘Galega Vulgar’ and cv. ‘Arbequina’) were successfully used as initial explants to establish and maintain high- and low-efficiency somatic embryogenic lines. Callogenesis was achieved in all explant types, with cv. ‘Galega Vulgar’ exhibiting higher *calli* formation in radicles and distal cotyledons. Similar results were reported by Pires et al. [31] in the same cultivar, in which radicles and distal cotyledons showed the highest callogenesis rates under light conditions. In cv. ‘Arbequina’, no significant differences in callogenesis were observed between radicle and cotyledon explants. These findings are consistent with those previously described in the same cultivar [49] but differ from the results of Trabelsi et al. [50], who, despite working with cv. ‘Arbequina’, reported lower callogenesis rates than those obtained in the present study.

Rhizogenesis was observed in all explant types from both cultivars, with higher potential in *calli* derived from radicles. In this study, cv. ‘Arbequina’ showed significantly higher rhizogenesis rates than cv. ‘Galega Vulgar’, regardless of explant type. These results align with Trabelsi et al. [50]. Overall, rhizogenic response appears to be genotype-dependent, with cv. ‘Arbequina’ showing greater morphogenic potential. As reported by Orinos and Mitrakos [51], this response is typical of juvenile or immature tissues, particularly under auxin-based conditions. The formation of adventitious roots during SE induction from zygotic embryos is an expected phenomenon in plant tissue culture, as reported in several plant species, including *Malus domestica* [52] and *Rosa hybrida* [53].

Regarding embryo formation, all explant types responded positively to the tested protocol, with radicles showing the highest response. The outstanding reaction of radicles for SE initiation, compared to cotyledons, has been previously reported in the cvs. ‘Koroneiki’ [54], ‘Zard’ [55], and ‘Picual’ [28]. Peyvandi et al. [54], in a study of the cvs. ‘Zard’ and ‘Mission’, observed that embryogenic potential decreases from the radicle toward the proximal regions of the embryo. This phenomenon is likely due to the higher density of young and competent cells in radicles, which exhibit greater potential for dedifferentiation and redifferentiation into somatic embryos [15]. Additionally, the hormonal balance between auxin and cytokinin in radicles appears more suitable for SE initiation than in cotyledons [56].

The results presented here confirm that embryogenic capacity can be sustained over successive cycles of secondary SE, enabling the identification of somatic embryogenic lines with different abilities to differentiate somatic embryos. Cyclic embryogenesis from zygotic embryos in olive has previously been achieved in several genotypes, as exemplified by studies conducted on the cv. ‘Picual’ [28,45,57] and cv. ‘Dahbia’ [29] using ECO gelled medium. Liquid embryogenic cultures are widely employed to promote homogeneous cell proliferation [58] and to facilitate the investigation of secreted biomolecules [59,60]. In olive, our previous research aimed at establishing a methodological framework for the isolation and characterisation of proteins and metabolites associated with SE, underscoring the significance of this system by revealing a high diversity of secreted compounds in liquid embryogenic cultures [60]. However, despite its advantages, the maintenance of embryogenic cultures in liquid medium presents considerable challenges, primarily due to the elevated risk of contamination. To address this limitation, cultures in the present study were initially propagated on ECO gelled medium to enhance proliferation, followed by transfer to liquid medium to mitigate the risk of culture loss.

Low-efficiency lines exhibited higher levels of phenolic oxidation and reduced proliferation compared to high-efficiency lines. These characteristics were evident from the first subculture on ECO gelled medium and persisted throughout the experiments. Moreover, the low-efficiency lines were more difficult to maintain in a proliferative state, and some embryogenic lines did not respond to the inoculation in liquid medium. Previous studies developed with the cv. ‘Chemlal’ [27] reported similar results, highlighting the occurrence of phenolic oxidation from the first subculture, leading to tissue necrosis and consequent loss of some lines. This oxidative response may be associated with stress-induced metabolic activity, as previously reported [59,61,62]. Stress response proteins, such as peroxidases, catalases, and superoxide dismutase, are known to regulate several physiological processes, including de novo organogenesis and lignification [63,64], and have been proposed as biochemical markers. A few of these tissue-level indicators, specifically peroxidases, were also found in the secretome by Pires et al. [60]. In addition, the authors reported the detection of alpha-amylase, an enzyme that plays a key role in the breakdown of starch into simple sugars. Its activity has been associated with providing an energy source for root and shoot development during the conversion of somatic embryos [65]. Its accumulation in mature somatic embryos has been documented in *Musa* spp. [66] and *Araucaria angustifolia* [67], indicating active starch mobilisation and its role as an energy source during somatic embryo germination. In addition, the identification of alpha-amylase in the secretome of a highly competent embryogenic line may indicate its presence in the embryogenic *calli*, consistent with the detection of starch granules in high-efficiency lines revealed by histological analysis. This observation supports the hypothesis of active carbohydrate metabolism, in which alpha-amylase contributes to the hydrolysis of starch into simple sugars, enabling the mobilisation of energy reserves to sustain embryogenic development. Additionally, embryos from high-efficiency lines showed a greater capacity for plant regeneration than those from low-efficiency lines, as shown by somatic embryo conversion assays. This improved performance may be associated with more effective starch mobilisation, as evidenced by the presence of starch granules and the involvement of alpha-amylase in maintaining energy supply during embryo germination.

Because prolonged subculturing in SE systems has been linked to the induction of somaclonal variations [68], it is crucial to assess the genetic stability of in vitro protocols before their application. In this context, flow cytometry was employed to evaluate chromosomal stability in embryogenic *calli*, somatic embryos, and regenerated plants. Genetic and epigenetic alterations in somatic cells during multiple mitotic divisions of a single clonal explant can result in somaclonal variations [69]. Such variations have been identified using cytogenetic techniques, such as flow cytometry, in several plant species, including *M. acuminata* [38], *V. vinifera* [70], *Piper hispidinervum* [71], and *Camelina sativa* [72]. Data revealed a high proportion of samples with normal ploidy levels. However, instances of aneuploidy and abnormal embryo development were observed. Bradaï et al. [39] investigated the effects of genotype and culture age on somaclonal variation in olive plants regenerated via SE from seeds of the cv. ‘Picual’ using morphological and biometric analyses of vegetative and reproductive traits. Their findings demonstrated significant somaclonal variation, with biometric analyses revealing intraclonal differences in the development of quantitative traits. Genotype was identified as a critical factor, with older lines exhibiting higher variability [73]. Somaclonal variation in SE-derived plants was confirmed by RAPD and SSR markers analysis, with older lines showing greater genetic instability, primarily attributed to genotype dependency.

The present study highlights the influence of genotype on the morphogenic response, with cv. ‘Arbequina’ exhibited a higher SE potential across all explant types. Embryogenic cultures from both cultivars remained stable in liquid medium across successive subcultures. The availability of lines with different embryogenic potential enables further research on the role of biomolecules secreted into the culture medium. Although the genetic stability of the cultured lines was not a primary goal of this study, the findings underscore the importance of testing the genetic uniformity of plants derived from SE whenever this technique is used in olive for clonal multiplication.

## 4. Materials and Methods

### 4.1. Plant Material

Mature olive fruits from the cv. ‘Galega Vulgar’ and cv. ‘Arbequina’ were collected after complete ripening, in November, from 12-year-old plants in open-pollinated orchards located at the Coleção Nacional de Referência de Cultivares de Oliveira—Instituto de Investigação Agrária e Veterinária (Elvas-Portugal). The cv. ‘Galega Vulgar’, a traditional Portuguese cultivar known for its high-quality olive oils, is recognised for its recalcitrance in morphogenetic responses, particularly its low capacity to develop adventitious roots. In contrast, the cv. ‘Arbequina’, a Spanish cultivar that is well adapted to intensive orchard systems, is characterised by high rooting competence [74]. Since adventitious rooting is a morphogenic process responsive to stress, we compared the embryogenic potential of the two genotypes.

The pulp was removed from the fruits, and the seeds were collected, washed, and stored at 4 ± 1 °C for 30 days. Afterwards, the endocarp was then broken using a manual press, and the seeds were placed in sterile water for 42 h in the dark at 24 ± 1 °C. Seeds were disinfected by immersion in 70% ethanol for 30 s, followed by a 20 min treatment with 10% sodium hypochlorite solution containing 0.1% Tween-20 under agitation, and rinsed three times with sterile bi-distilled water. Under aseptic conditions, radicles and cotyledons (proximal and distal) were excised and used as initial explants, following the procedure previously described by Pires et al. [31].

**Figure 9:**
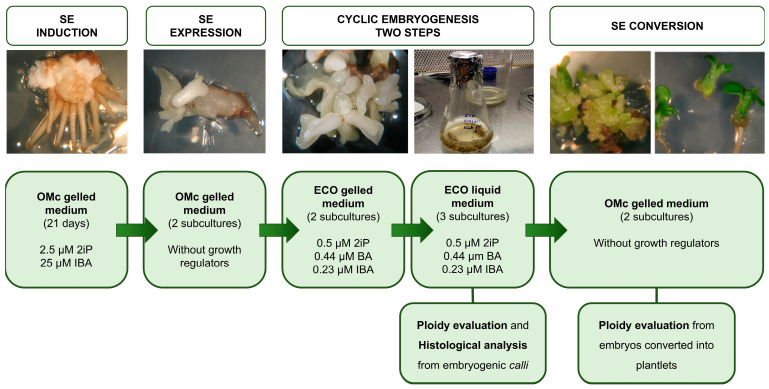
Schematic representation of the workflow, illustrating the four phases of SE: induction, expression, maturation, and conversion. Additionally, cyclic embryogenesis is presented as a key procedure for the multiplication and maintenance of embryogenic material.

Figure 9 illustrates the entire process from the induction phase through to the analysis of embryogenic competence and the evaluation of conversion performed for each embryogenic line, highlighting the key steps and methodologies employed.

### 4.2. Establishment and Development of Somatic Embryogenic Cultures

Embryos’ radicles and cotyledons, proximal and distal (measuring approximately 0.8 mm and 3.4 mm, respectively, in cv. ‘Galega Vulgar’, and 0.1 mm and 0.5 mm in cv. ‘Arbequina’), were excised from previously disinfected seeds, and inoculated in Petri dishes (7 cm Ø) containing induction OMc culture medium [51], supplemented with 2.5 µM 6-dimethylallylamino-purine (2iP), 25 µM indole-3-butyric acid (IBA), and gelled with 6 g L^−1^ Agar-Powder (VWR, Portugal). The pH was adjusted to 5.8 prior to autoclaving. The three types of explants (radicle, proximal cotyledon, and distal cotyledon) taken from a single seed were inoculated per Petri dish. Explants taken from a total of 134 and 164 seeds were considered for the cv. ‘Galega Vulgar’ and the cv. ‘Arbequina’, respectively. Cultures were kept at 25 ± 1 °C in the dark for 21 days.

After the induction phase, explants were transferred to OMc gelled medium devoid of growth regulators to promote embryo differentiation, henceforth called expression medium. The cultures were maintained for two months under the same growth conditions as described above, with two subcultures lasting 30 days each.

### 4.3. Establishment of Cyclic SE in Liquid Medium

To promote cyclic embryogenesis, the embryogenic lines were subcultured into ECO (embryogenesis cyclic olive) gelled medium [57,75] supplemented with 50 mg L^−1^ myo-inositol, 550 mg L^−1^ l-glutamine, 0.5 µM 2iP, 0.44 µM 6-benzyladenine (BA), 0.23 µM IBA, 20 g L^−1^ sucrose, and 1 g L^−1^ casein hydrolysate, and gelled with 2.5 g L^−1^ Gellan Gum^®^. The pH was adjusted to 5.8 before autoclaving. Cultures were maintained at 25 ± 1 °C in the dark and subcultured twice, each subculture lasting 30 days. After this period, approximately 200 mg of embryogenic *calli* was transferred into 100 mL Erlenmeyer flasks containing 50 mL of ECO liquid medium with the same composition. To evaluate the embryogenic capacity of the somatic embryogenic lines, *calli* were collected by filtration at the end of each subculture period, and the number of differentiated somatic embryos was recorded. This culture approach promoted both the stabilisation and proliferation of embryogenic lines under consistent conditions, enabling the production of sufficient plant material while preserving genetic variability before classification based on SE efficiency. This strategy also facilitates procedures aimed at characterising secreted biomolecules involved in SE efficiency, aligning with the broader objectives of the ongoing research [60].

### 4.4. Conversion of Somatic Embryos

At the end of the third subculture in ECO liquid medium, after approximately 7 months from the beginning of the assay, embryogenic material was collected and transferred to Petri dishes containing OMc gelled expression medium. Embryogenic efficiency was expressed as the number of somatic embryos developed per gram of embryogenic tissue, thirty days after *calli* establishment. For this purpose, a total of 15 segments, each approximately 200 mg, were collected from the *calli* of each embryogenic line from both cultivars and inoculated into Petri dishes (9 cm Ø) containing ~20 mL of OMc expression medium. Each *calli* segment was considered as an independent biological replicate. The cultures were maintained at 25 ± 1 °C under a 16 h photoperiod, with a light intensity of 40–45 µmol m^−2^ s^−1^, for 30 days. The data were subjected to statistical analysis to assess differences in embryogenic potential among lines within each cultivar.

To determine the optimal developmental stage for converting somatic embryos into plants, previously characterised embryos from high- and low-efficiency lines were divided into two morphological stages: globular and torpedo. Thirty days after the establishment of embryogenic *calli* under expression conditions, embryos identified at two developmental stages were isolated and vertically inoculated into Petri dishes (9 cm in diameter) containing fresh OMc gelled expression medium. A total of 488 somatic embryos were established (266 globular and 222 in the torpedo stage). A total of 20 embryos were inoculated per Petri dish, which served as a biological replicate (a minimum of six replicates were considered per embryo developmental stage). The cultures were maintained for 30 days under the same temperature, photoperiod, and light intensity conditions as previously described. At the end of the subculture, the converted somatic embryos were transferred to test tubes containing OMc gelled expression medium supplemented with 1 g L^−1^ activated charcoal. The embryos were maintained at 26 ± 1 °C, with a 16 h photoperiod and 100 µmol m^−2^ s^−1^ of light intensity, for two months. Obtained plants were further subjected to an acclimation procedure before being transferred to greenhouse conditions.

Plantlets were transferred to polypropylene honeycomb trays containing a mixture of sand, perlite, and peat in the proportion of 1:1:3 (*v*/*v*). To prevent dehydration, the trays were kept in stalls with a transparent plastic polyethene cover. Plants were maintained under controlled conditions in a plant growth chamber with 24 °C/22 °C day/night, 60% humidity, 16 h photoperiod and light intensity of 100 µmol m^−2^ s^−1^. After 15 days, the covers were removed, and plants remained under the same conditions for an additional 15 days. Finally, the plants were transferred to a greenhouse.

### 4.5. Histological Analysis

To assess differences in tissue microanatomy between high- and low-efficiency embryogenic lines, histological analyses were conducted. At the end of the third subculture in ECO liquid medium, three segments each of high- and low-embryogenic calli were randomly sampled and fixed in FAA solution (formaldehyde:acetic acid:70% ethanol, 1:1:8, *v*/*v*) for 48 h. The samples were further washed twice with 70% ethanol and subsequently dehydrated in a series of six solutions with increasing concentrations of butanol (10, 20, 35, 55, 75, and 95%), followed by two 30 min washes with xylene. Subsequently, impregnation was carried out by repeatedly submerging the samples in liquid paraffin wax at 58 °C over a total period of 56 h, with the immersion process performed three times in total. To achieve the paraffin wax blocks, the samples were embedded into liquid paraffin (at 58 °C), which was further left to cool in a freezer platform. The blocks were maintained in the freezer until further processing. Sections of 5–10 μm thickness were cut on a rotative microtome (Leica Reichert-Jung 2045, Wetzlar, Germany) and assembled on glass slides. Following slide preparation, the sections were subjected to a staining protocol. Paraffin was removed by two 5 min. washes in xylene, after which the samples were rehydrated in an ethanol series (100%, 95%, and 70%) and placed in distilled water for 5 min. A double staining procedure was then performed according to Sass [76]. Tissues were initially immersed in an aqueous solution of Safranin O (0.01%, *w*/*v*) for 1 min to stain chromosomes, nuclei, and lignified cell walls. This was followed by rinsing with distilled water to remove excess stain. The slides were subsequently dehydrated through an ascending ethanol series (70% and 95%), stained with Fast Green FCF (0.5%, *w*/*v* in 95% ethanol) for 5 s to visualise the cytoplasm, and washed thoroughly in absolute ethanol. Finally, the slides were cleared with two 2 min washes in xylene, dried, and mounted with DPX mounting medium for histology (Sigma-Aldrich, Madrid, Spain). Histological observations were made using an automated microscope (Leica DM6000B, Wetzlar, Germany), and images were captured with a camera (Leica CC7000, Wetzlar, Germany).

### 4.6. Evaluation of Ploidy Variation Through Flow Cytometry

To assess the presence of somaclonal variation associated with ploidy changes, a total of 45 samples were analysed using flow cytometry. These included somatic embryos with normal and abnormal morphology, embryogenic *calli* with high- and low-efficiency, and leaves from plants derived from somatic embryo conversion. Flow cytometry analysis was conducted at Plant Cytometry Services (The Netherlands, Didam) following the protocol of Arumuganathan and Earle [77]. Briefly, approximately 50–100 mg of fresh plant tissue was finely sectioned with a sharp razor blade in an ice-cold DNA buffer to isolate cell nuclei. As an internal standard, fresh tissue from a reference plant was simultaneously sectioned with the olive samples in a DNA buffer (stored at 4 °C) composed of 5 mM HEPES, 10 mM magnesium sulfate heptahydrate, 50 mM potassium chloride, 0.2% Triton X-100, 0.1% dithiothreitol, 1.0% PVP-40, and 2 mg L^−1^ DAPI, with the pH adjusted to 7.5 [78]. The DAPI fluorescent dye selectively complexes with double-stranded DNA to give a product that fluoresces at 465 nm. DAPI has specific DNA-binding properties with a preference for adenine–thymine (AT)-rich sequences. Further, the buffer containing cell constituents and large tissue fragments was passed through a nylon filter of 50 µm mesh size.

The DNA content of the stained nuclei was measured after incubation using a Sysmex CyFlow Ploidy analyser with a UV high-power LED (365). The fluorescence of the stained nuclei, passing through the focus of a light beam from a high-pressure mercury lamp, was measured by a photomultiplier and converted into voltage pulses. These voltage pulses are electronically processed to yield integral and peak signals and can be processed by a computer. A diploid *A. schoenoprasum* sample (2n = 2x = 32; 2C DNA amount = 33.50 pg) was used as the internal standard. The ratio between the mean fluorescence of G0/G1 nuclei of the olive sample and the internal standard *A. schoenoprasum* was calculated as an indicator of the ploidy level of each sample.

### 4.7. Statistical Analysis

Statistical analyses were performed using SPSS version 28.0. Normality and homogeneity of variances were assessed using the Shapiro–Wilk and Levene’s tests, respectively. Comparisons of mean somatic embryo conversion rates at different developmental stages, between high- and low-efficiency embryogenic lines and between the two cultivars, were conducted using the Student’s *t*-test or the nonparametric Mann–Whitney U test when parametric assumptions were not met. For callogenesis, rhizogenesis, necrosis, and SE parameters, mean comparisons among different explants within each cultivar were performed using one-way ANOVA (followed by Tukey’s HSD test) or the Kruskal–Wallis test when parametric assumptions were not met. The number of somatic embryos from embryogenic lines in both cultivars, across three subcultures in ECO liquid medium and one subculture in OMc gelled expression medium, was analysed using one-way ANOVA (followed by Tukey’s HSD test) or the Kruskal–Wallis test when appropriate. Statistical significance was set at *p* < 0.05.

## 5. Conclusions

To the best of our knowledge, in vitro cultured lines with differentiated embryonic capacity were, for the first time, maintained with systematic behaviour in olive in a process of cyclic SE. This was achieved using a comprehensive protocol for SE, based on zygotic embryos from the cvs. ‘Galega Vulgar’ and ‘Arbequina’, as initial explants. Acquired data allowed us to confirm genotype dependency in the success of somatic embryo production within the same species. The use of the ECO culture medium enabled the maintenance of embryogenic *calli* without altering the efficiency of embryogenic lines across multiple subcultures. Regarding somatic embryo conversion, it was possible to conclude that the globular and torpedo stages are the earliest and most effective stages for converting somatic embryos into plants in all cultivars. However, the conversion efficiency is also influenced by the genotype and the embryogenic line, with high-efficiency lines showing better embryo plant conversion rates than low-efficiency lines. Chromosomal stability was evaluated by flow cytometry, revealing a high degree of genetic stability in embryogenic *calli*, somatic embryos and regenerated plants across all studied somatic lines. Nevertheless, some situations of aneuploidy were detected, confirming the need for the genetic evaluation for all individuals obtained by SE whenever the technique is used for clonal propagation in olive. The availability of both high and low somatic embryogenic lines will enable further research focused on evaluating differences in biomolecules secreted into the culture medium. This will provide insights into the molecular mechanisms underlying SE recalcitrance and guide future studies aimed at elucidating the processes involved in SE signalling. Considering the importance of these biomolecules in cellular communication, we hypothesise that they may play a crucial role in understanding SE recalcitrance in certain genotypes.

## Figures and Tables

**Table 1:** Callogenesis and rhizogenesis rates (%) obtained during the induction phase, along with necrosis and somatic embryogenesis (SE) rates observed during the expression phase, are presented for all explant types (radicles and proximal and distal cotyledons) in both cultivars. Different lowercase letters indicate statistically significant differences (*p* ≤ 0.05) among the three explants (radicle, proximal cotyledon, and distal cotyledon) within a cultivar, while uppercase letters denote significant differences between cultivars for the same explant type. Data are expressed as mean ± standard error.

Cultivar	Explant		Induction		Expression	
		*n*	Callogenesis	Rizhogenesis	Necrosis	SE
‘Galega Vulgar’	Radicle	134	48.5 ± 4.3 Ab	9.7 ± 2.6 Ab	29.1 ± 3.9 b	8.2 ± 2.4
	Proximal		27.6 ± 3.9 Aa	0.8 ± 0.7 Aa	16.4 ± 3.2 Aa	2.2 ± 1.3
	Distal		39.6 ± 4.2 Aab	0.8 ± 0.7 Aa	21.6 ± 3.6 Aab	4.5 ± 1.8
	Radicle	163	65.6 ± 3.7 B	30.1 ± 3.6 B	34.4 ± 3.7	9.8 ± 2.3 b
‘Arbequina’	Proximal		66.8 ± 3.6 B	21.5 ± 3.2 B	33.1 ± 3.7 B	1.9 ± 1.1 a
	Distal		66.9 ± 3.7 B	22.7 ± 3.3 B	30.1 ± 3.6 B	4.9 ± 1.7 ab

## Data Availability

The raw data supporting this study are included in the article. Further inquiries can be directed to the corresponding author.

## Abbreviations

The following abbreviations are used in this manuscript:

SE	Somatic Embryogenesis
ECO J	Embryogenesis Cyclic Olive
OMc	Olive Medium Culture
IBA	Indole-3-Butyric Acid
BA	6-Benzyladenine
2iP	6-Dimethylallylamino-Purine
